# Device for automatic measurement of light pollution of the night sky

**DOI:** 10.1038/s41598-022-20624-7

**Published:** 2022-10-01

**Authors:** Dominika Karpińska, Mieczysław Kunz

**Affiliations:** Department of Geomatics and Cartography, Faculty of Earth Sciences and Spatial Management, Nicolaus Copernicus University in Toruń, Toruń, Poland

**Keywords:** Environmental sciences, Techniques and instrumentation, Urban ecology, Environmental social sciences

## Abstract

Research on light pollution of the night sky has been carried out in Toruń, Poland since 2017. Initially, the measurements were conducted within a network of 24 points using a handheld sky quality meter with lens (SQM-L) photometer (Unihedron, Canada). Based on these measurements, the first accurate maps of night sky pollution by artificial light in Toruń have been developed, both in seasonal and annual terms. Using the experience gained and elements of modern technology, a decision was made to construct an automatic network of mobile devices measuring light intensity at night, covering the entire city of Toruń. This paper presents the technical characteristics of the constructed automatic measurement devices that make up the distributed monitoring network and the process of testing and using the devices. The implementation of this project has started in 2020. To accommodate the evolving expectations of different user groups and the observed trends in the concept of Smart Cities, especially those related to the communication between devices of the Internet of Things, LoRaWAN was selected for data transmission. The first stage involved the construction of a prototype of an automatic, portable and cost-effective device, which was subjected to months of field testing under operational conditions. The device was built using off-the-shelf electronic components and a housing that met the requirements for outdoor use. The next stage was to calibrate the device by simultaneously comparing the obtained results with measurements taken using professional SQM devices. This was followed by the preparation of 35 identical devices, which are already operating in the measurement network in the city of Toruń. Elements of the network are prepared in a way that allows for further expansion and makes data available in the form of an application for many recipients.

## Introduction

Human actions in the geographical environment transform the last natural ecosystems and further modify previously altered ones. The observed transformations relate to the timing of the factor, its spatial extent and the scale of impact^1,2^. Changes in the environment, their direction and, above all, their intensity, as well as, in some situations, irreversibility of the transformations, have become one of the main reasons for the search for new, effective research methods and for an increased interest in comprehensive monitoring of the key environmental parameters, as well as for strengthening the influence and the role of education and the importance of environmental awareness.

People are becoming increasingly conscious of the impact and consequences of their actions on the state and quality of the environment, especially in the vicinity of their homes and immediate surroundings. They are also increasingly aware of long-term threats and their negative impact on the life, health and functioning of all living organisms. There is growing recognition of the issue of smog and air pollution by particulate matter or concentrations of gases, water and soil by heavy metals and pesticides, radioactive contamination, noise pollution, odour, and increasing light pollution of the night sky^2–11^.

The last mentioned phenomenon is caused by excessive emission of artificial light at night, mainly by inappropriately mounted street lamps, illumination of places that should not be lit, and the use of poorly designed light illuminations^9^. About 80% of the world's people and as much as 99% of European and US citizens live in light polluted areas^12^. Excess of this factor can cause hormonal disorders in humans, including melatonin deficiency resulting in problems with metabolism and sleep. The negative consequences are also visible in the development of plants and animals that are used to the circadian rhythm in evolution^13^.For many decades, research has been conducted on the impact of these phenomena on human health and the quality of life, resulting in the search for effective ways to reduce or eliminate them. Monitoring of selected environmental parameters is carried out in several different ways and the results are made public in a number of forms. Several so-called instrumental and analytical methods are available, which are widely used to determine the size and extent of the degradation factor. Analysis of various types of pollution is nowadays a thriving part of science involving chemistry, environmental sciences, biology and other natural and technical sciences. Monitoring of a global phenomenon has become an interdisciplinary issue, and thanks to the development of technology, it is now highly automated. To determine environmental parameters, methods are used that involve both simple measurements and complex laboratory tests requiring advanced knowledge and equipment^2^. The majority of phenomena negatively affecting the environment are usually subject to spot measurements spread over time. Nevertheless, monitoring networks are becoming increasingly widespread, thanks to which it is possible to determine both the extent and magnitude of a phenomenon and its course and variability in time and space. These efforts enable a more thorough understanding of a harmful process, its course, distribution, impact on the immediate and distant surroundings, as well as on the life and health of living organisms, from plants, through animals to humans. Multi-faceted knowledge of an observed phenomenon allows us to take more effective countermeasures and create an effective mechanism to mitigate its impact. One such example is research on the occurrence of airborne particulate matter. Nowadays, remote measurements and monitoring with real-time assessment of air quality has become standard in most cities^2,14^. Through this type of research, it has been possible to introduce a number of measures to prevent or reduce negative effects, as well as to inform the public about the current level of risk.

This paper describes an automatic device constructed by the authors to measure the pollution of the night sky by artificial light, a phenomenon that is increasingly observed, universally recognised and of interest to a growing group of scientists from many countries^11,15–19^. Various measurement methods have been developed to understand and determine the scale of the phenomenon. In technical terms, they can be divided into instrumental and observational methods, whereas from the functional point of view into those that can be used only by a qualified operator or by an amateur with no professional knowledge^15,17,20,21^. The most commonly used methods of measuring the light pollution are those using photometers and digital cameras^3,15,16,19,20^. Measurements made in this way are standardised, thus it is possible to compare the intensity of the phenomenon at different measurement sites located all over the world^14,20,22^. Research with the use of satellite imagery has also being carried out on an increasingly large scale. For this purpose, images taken by, e.g. the Suomi NPP satellite with the visible/infrared imaging radiometer suite (VIIRS) Day/Night Band instrument, the Defense Meteorological Satellite Program (DMSP) satellite with the operational linescan system (OLS) instrument, and Luojia 1-01 satellites are used^23,24^. Models of night sky brightness have also been developed^12^, making it possible to predict brightness values at any location around the globe.

## Wireless network

The development of environmental monitoring would not be possible without continuous technological progress, public pressure and targeted environmental education. To meet the expectations of users, measuring instruments must not only be ever more accurate, but also practical and straightforward to use and economical to operate. Environmental analytics has begun to strongly overlap and interpenetrate with broadly defined modern digital technology and informatisation^25,26^. Nowadays, creating new measurement solutions is an interdisciplinary challenge for representatives of both natural and technical sciences, including computer scientists and programmers. In the implementation of largescale measurements of spatially distributed phenomena, fully mobile devices become indispensable, as they transmit the collected data to a server where they are analysed, shared, and often visualised. This will also allow the collected measurement data from ever longer observation sessions to be archived and utilised.

The development of a fully mobile, and thus highly functional recording device anticipated by the target market is accomplished through the use of wireless data transmission technology. Depending on the needs, such technologies can be divided related to the range of data transmission and the transfer size. Each of these technologies is characterised by a specific subset of users, as well as parameters and technical requirements. The most popular long-range data transmission technologies include the widespread GSM network, while Wi-Fi or Bluetooth networks are characterised by a much shorter range of up to several dozen metres^27,28^. In terms of data packet transmission rate, on the other hand, GSM and Wi-Fi networks can transmit significantly more data than Bluetooth. When it comes to environmental measurements implemented by a distributed sensor network, the most important operational parameter, which determines the choice of a given solution, is the power consumption used when sending messages. The raw measurement data will usually contain relatively small-sized information packets, but their deployment in areas with difficult access to power supplies makes it necessary to use a solution based on its own energy source, usually a battery with a long or very long life without the need for frequent replacement or charging. The abovementioned wireless data transmission technologies are too energy-intensive for such tasks, as they consume a significant amount of energy during data transmission, which prevents long-term monitoring of environmental data on a larger spatial scale and in uninhabited areas or areas with limited technical infrastructure. LPWAN (Low Power Wide Area Network) networks have been designed with such applications and peripheral locations in mind^29,30^. Due to its extensive applicability and parameters, LPWAN is one of the most modern technologies increasingly used for the communication between devices^30–33^. As one of the many elements of Industry 4.0, it fits into the idea of internet of things (IoT), supporting Smart City solutions. Of the available types of LPWAN networks, three are most extensively used: Sigfox, NB-IoT and LoRaWAN. Each of these types of solutions has its own parameters and is intended for different applications^30,33^.

**Figure 1:**
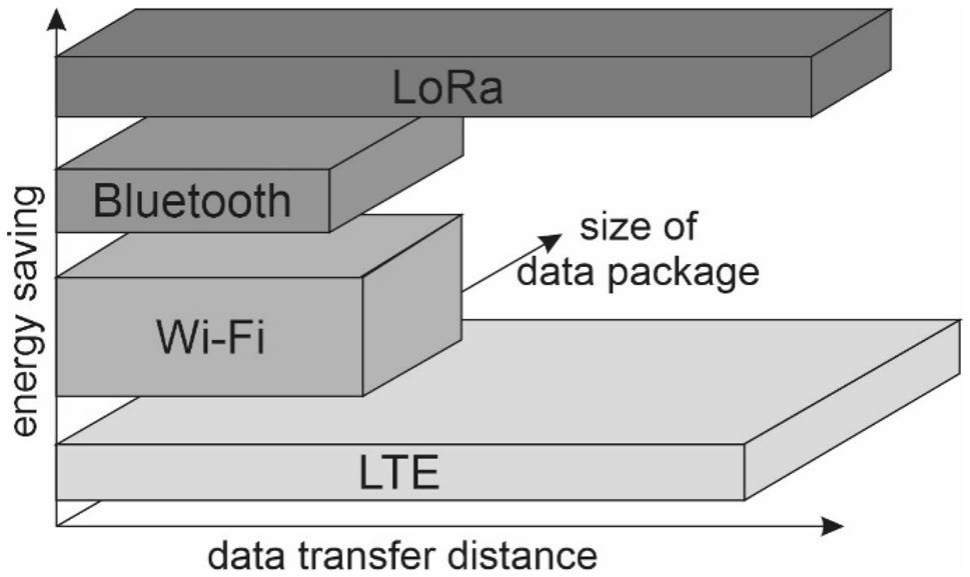
Schematic comparison of selected parameters (data transmission distance vs. energy efficiency) of the most popular wireless technologies on the market.

The LoRaWAN standard was selected for the implementation of this project involving the monitoring of night sky pollution by artificial light. It has the most optimal parameters in relation to the prepared design objectives and applications of the designed device. The LoRaWAN standard is one of the MAC (Medium Access Control) radio communication protocols^28,34,35^ and is characterised by a long range allowing network connectivity with low power consumption. LoRa technology is used for communication in the LoRaWAN standard and is, for such applications and with the input limitations indicated, an alternative to other technologies such as LTE, Wi-Fi or Bluetooth (Fig. 1).

LoRa is a type of modulation that uses the CSS (Chirp Spread Spectrum) technique, consisting in spreading the spectrum of the transmitted signal^29,33–35^. The CSS modulation makes full use of the allocated transmission bandwidth, which increases the robustness of the communication against interference, and eliminates inaccuracies related to the Doppler effect and route propagation. LoRaWAN is developed as an open standard that uses the ISM (Industrial, Scientific, Medical) radio spectrum and requires no licence fees. In Europe, LoRa operates at the 868 MHz frequency band. The great advantage of the selected technology is its range, which in the field conditions varies, depending on the type of housing development, from several hundred metres to several kilometres^28,33,36^. In the professional literature, one can find examples of measurements and data transmission performed under specific conditions, over a distance of up to 702 km^28^. An additional key function of LoRaWAN is the possibility of bidirectional communication, allowing not only to send but also to receive information. In the process of planning a wireless network, the advantage of LoRaWAN is the use of an unlicensed radio band, thanks to which there are no restrictions and additional requirements, or costs associated with the activation and operation of an already established network. In practice, the LoRaWAN architecture consists of four main components, such as end devices (data loggers), communication gateway(s) and a network and application server.

Due to its universal parameters, LoRaWAN technology is used in many fields of application. Such solutions can be found, among others, in defining the objectives of Smart City, where they form the basis for the transmission of traffic, environmental and logistic information, in the construction industry in monitoring the status of operation and quality of structures, and in modern medicine when monitoring the health of patients staying outside medical facilities^25,32,35,37^.

## Methodology of work on device for remote measurement of night sky light pollution

For several years, systematic research has been carried out on the pollution of the night sky by artificial light in the city of Toruń^11,36,38^. The main objective is to monitor this phenomenon, including its spatial and temporal variability and the most important factors affecting it. Based on previous experience, an in-house measurement device was constructed to automate the process of data acquisition.

### Genesis of the project

The first measurements pertaining to the phenomenon of night sky pollution in Toruń were made in autumn 2017, followed by regular observations using a handheld SQM photometer (Unihedron, Canada) as part of a project implemented in 2017–2018. To this end, a permanent measurement network distributed throughout the city was established, consisting of 24 locations. During a one night measurement session taking place during the astronomical night (there is no such period at the latitude of Toruń in the summer), sky brightness was measured at all sites. The results of spot monitoring were plotted using interpolation methods and visualisation tools available in GIS systems^11,39^, which helped to determine the spatial distribution and extent of night sky light pollution. The intensity of this phenomenon at each of the surveyed points was also explored in relation to the distinguished landcover categories and types of urban development.

Repeatable measurements performed regularly over such a long period of time were characterised by significant limitations. One measurement session was very time-consuming, as it lasted about two hours, during which time all measurement locations were visited, covering a distance of almost 50 km each night by car. Despite the observance of all time frames and sticking to the plan of fieldwork, measurements were not carried out simultaneously at all the locations, which affected the results, especially during the night with changing cloud cover. Although the measurements were carried out with great consistency and care, they were performed in a spatial buffer of about 5 m, which could unintentionally slightly affect the obtained results. An additional limitation was also a one-time night measurement at one point, instead of a whole series of measurements at specific time intervals. Inaccuracies in the readings within a single session could have been caused by sudden changes in meteorological parameters. In the adopted procedure, it was not possible to carry out simultaneous measurements in identical time and weather conditions at all the locations, not to mention the involvement of the personnel in each tour of the measurement network points.

Using the experience gained and after an analysis of the identified constraints and the technical capabilities at hand, work began in 2019 on developing a network for automatic remote monitoring of light pollution of the night sky in Toruń, based on designed in-house recording devices.

### Design, functional and utility features of the device

To enhance the research on light pollution in urban space, work has begun on the construction of a device that would perform automatic measurements, would be mobile, battery-powered and use long-range wireless communication. All the aforementioned features are in line with the strategy of Industry 4.0 and modern solutions proposed as part of the Smart City concept.

The concept of Industry 4.0 assumes the more and more common use of process automation as well as the processing and exchange of data with the use of new transmission technologies^26^. LoRaWAN is one of the solutions used for communication of Internet of Things (IoT) devices, which supports the development of Smart Cities in the Smart Environment area. As a result, the interactivity, frequency, and scope of measurements carried out in urbanized areas are increased^40,41^.

According to the developed project, the device was to serve as a meter of very low intensity light observed in the night sky. In this respect, it was necessary to use a sensor with technical parameters suitable for very accurate measurements of light intensity. To locally verify the weather conditions occurring during the operation of the device, it was decided to carry out additional simultaneous measurements of other environmental parameters—temperature and moisture content. The analysis of the spatial coverage of the study area indicated that 36 measurement devices should be deployed to provide full coverage of Toruń. The concept of creating an urban measurement network assumes the selection of points covering the whole city relatively evenly and representing different types of housing development and elements of land cover. It was assumed that measurements will be made only at night, between 21 p.m. and 6 a.m. on the following day, at 15 min intervals, and in addition, weather conditions will be recorded twice a day.

### Construction and technical parameters of the device, and selected characteristics of its components

A prototype device meeting all the predefined functionalities was constructed based on available electronic modules. The B-L072Z-LRWAN Discovery developmental board from STMicroelectronics^42^ was selected as the main electronic component providing wireless communication. This board has an integrated LoRa communication module, enabling low-power wireless messaging, and also allows the board to enter a low-power state during hibernation, and thus target long-term battery-powered operation. This module is fully programmable, which enables future expansion of the set with other functionalities. The TSL2591 light sensor from AMS, which has high sensitivity and registration accuracy, was selected as a component implementing the light intensity measurement. Its great advantage is a wide measurement range of 188 μlx to 88 000 lx, sensitivity reaching 0.000377 lx, and a wide dynamic range (WDR) of 600 M:1^43^. The sensor used has two diodes with different spectral properties. One of them registers visible light together with infrared (in the range from 400 to 1 100 nm), while the other is responsible for the registration of infrared light (between 500 and 1 100 nm). Thanks to this solution, we can use the results in various ways. The use of the formula provided by the manufacturer allows us to obtain spectral characteristics similar to the human eye. The presence of a compensating diode makes a difference compared to the sensor used in the SQM device, so the results obtained in the measurements may be slightly different.

To measure additional environmental parameters, the X-NUCLEO-IKS01A2 development board from STMicroelectronics was used, which is connected to the STM32 microcontroller via the I2C interface^44^. This board enables the recording of a number of parameters, however, in the constructed device it is only responsible for reading the temperature and humidity of the environment. This results from the necessity to limit the size of the message packets sent, while at the same time improving the operating range and reducing the power consumption of the device.

Once all the components had been selected, tested and integrated, the process of final connection and programming was carried out. The base of the device, i.e. the B-L072Z-LRWAN development board was connected to the X-NUCLEO-IKS01A2 environmental sensor board, using Arduino connectors. Using standard wires, a TSL2591 light sensor was added by connecting the corresponding I2C (SCL and SDA), power supply (VIN), sensor ground (GND) pins and the X-NUCLEO-IKS01A2 board.

**Figure 2:**
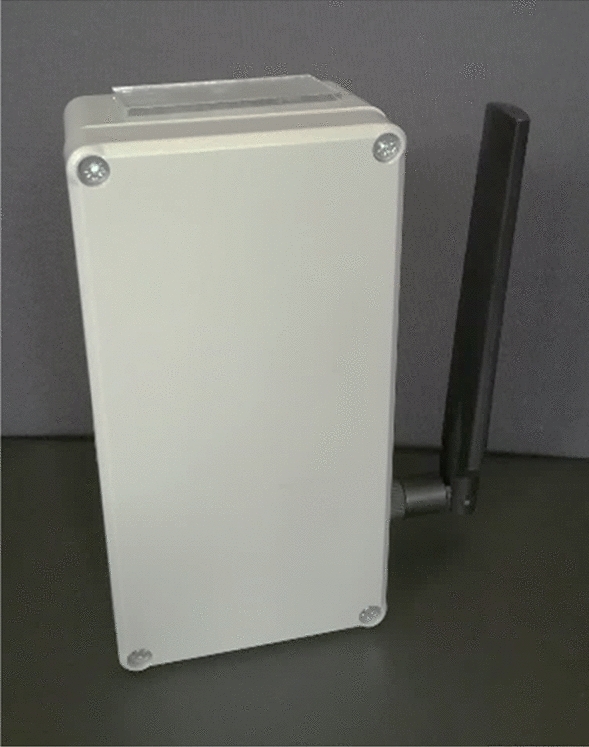
Constructed device view (photo by Dominika Karpińska).

All components used were placed in a standard external casing with dimensions of 8.0 × 5.4 × 15.8 cm. In its lower part an opening was made for an external antenna, while in the upper part a specifically selected opening was cut out, protected with a glass pane, through which measurements are performed by the light sensor (Fig. 2).

Following the above steps, an automatic device was constructed to record light intensity in the lower troposphere, i.e. to measure the pollution of the night sky by artificial light coming from the Earth’s surface. Selected technical parameters of the device are presented in Table 1.

**Table 1:** Selected technical parameters of the device for measuring light pollution of the night sky.

Parameter	Characteristic
Weight	380 g
Dimension	5.5 × 8.2 × 15.8 cm
Standard of data transmission	LoRaWAN
Frequency bands	868 MHz
Operating time [3 000 mAh]	~ 9 month
Range in built-up areas	3–4 km
Number of measurements during the day	36
Frequency of measurements	15 min
Operational time	21:00–06:00 CEST
Measuring sensors	light intensity, temperature, humidity
The half-cone angle of data collection	27°
Tightness class	IP65

### Flowchart of the system operation

**Figure 3:**
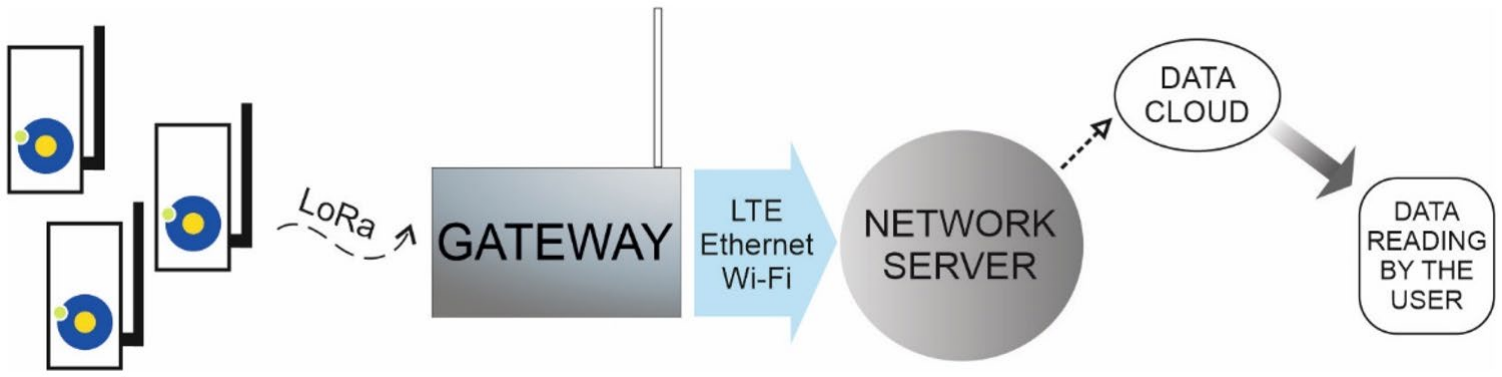
Schematic diagram of the measurement system.

After constructing the device and writing the control software, the construction of the entire measurement system was started. Each of the measuring instruments is ultimately connected to the communication gateway using LoRa technology. A MultiTech communication gateway with a LoRaWAN module was used as an access device. To successfully connect the gateway to the measuring device, it was necessary to configure the communication gateway software. To this end, the information about the unique device number (Dev EUI) and the application key and its number (App EUI and App Key) was used. Once the unit is configured, it is possible to send data to the communication gateway and read them using NodeRED, a programming tool where data are redirected to a selected server, which stores all measurement results. Figure 3 shows a schematic representation of the constructed measurement system.

## Test results for the measurement devices under operational conditions

**Figure 4:**
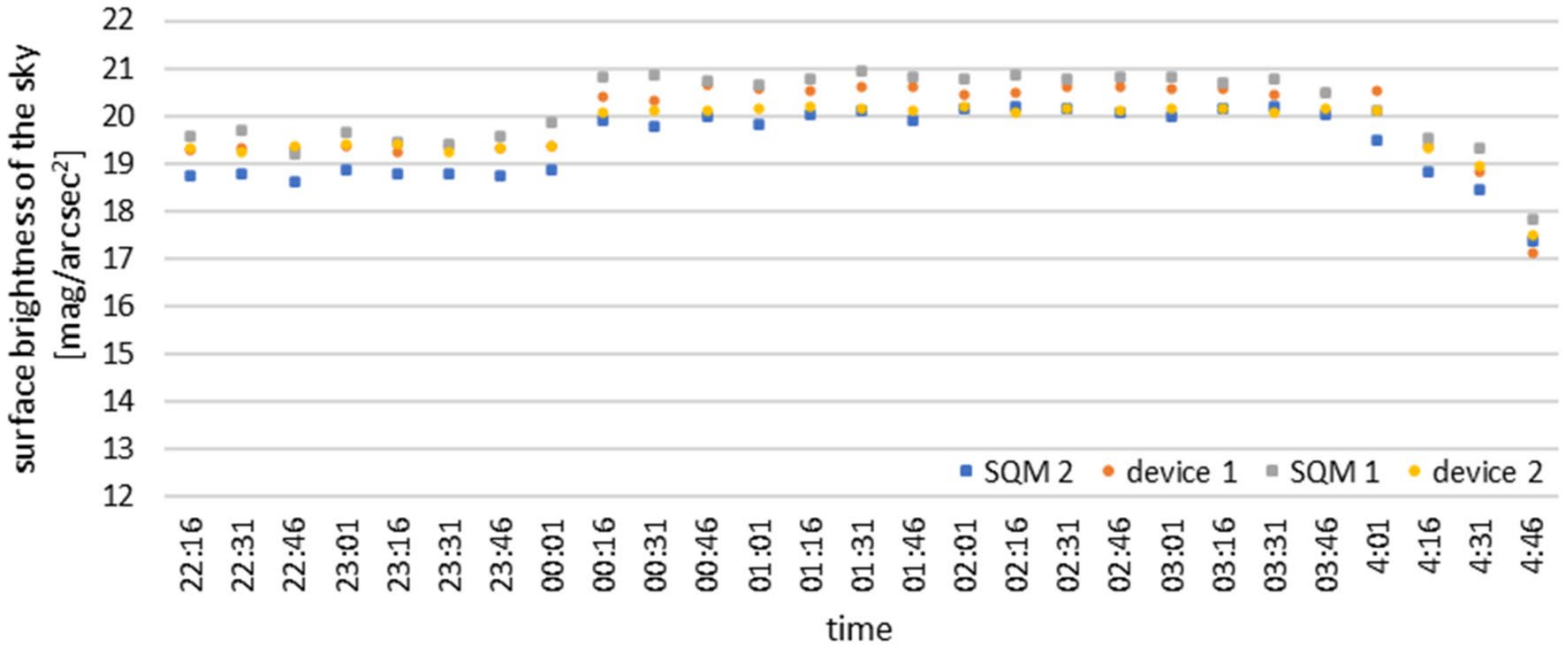
Comparison of the measurement results obtained by a device of its own design with the measurements made by SQM photometer (27–28.03.2020).

Following the construction of the prototype device and the preparation of all elements of the measurement network and their configuration, the necessary tests were carried out. The first of them was to check the correctness of the collected measurement data. For this purpose, two devices (1 and 2) were installed in close proximity to each other in the NCU premises, and the data collected were compared with measurements taken at the same time and place using a handheld SQM photometer version L. Sky brightness values recorded in lux were converted into units used in SQM photometers, by using a spherical angle and a commonly shared formula for converting to mag/arcsec^2^ units^18^. The obtained measurement results are presented in Fig. 4.

During the test measurements, the observed sky was cloudless. On the graph between the measurement by 0:01 and 0:16 you can see the change in the brightness of the sky, it is the result of turning off street lamps located on a nearby street. After 3:46 you can also see the rising Sun effect. The differences between the results are due to slightly different lighting conditions at the positions taken and from the possible non-vertical positioning of the manual SQM photometer used for the tests. Analysis of the collected data from the constructed devices and from the SQM photometer shows that they are comparable and overlap throughout the measurement period of 6 h. This proves that the digital light sensor used in the constructed device is of high accuracy and correctly performs the measurements in relation to the prevailing outdoor conditions.

### Technology demonstrator stage and comparison with the results from the 2017/2018 season (TARR location)

**Figure 5:**
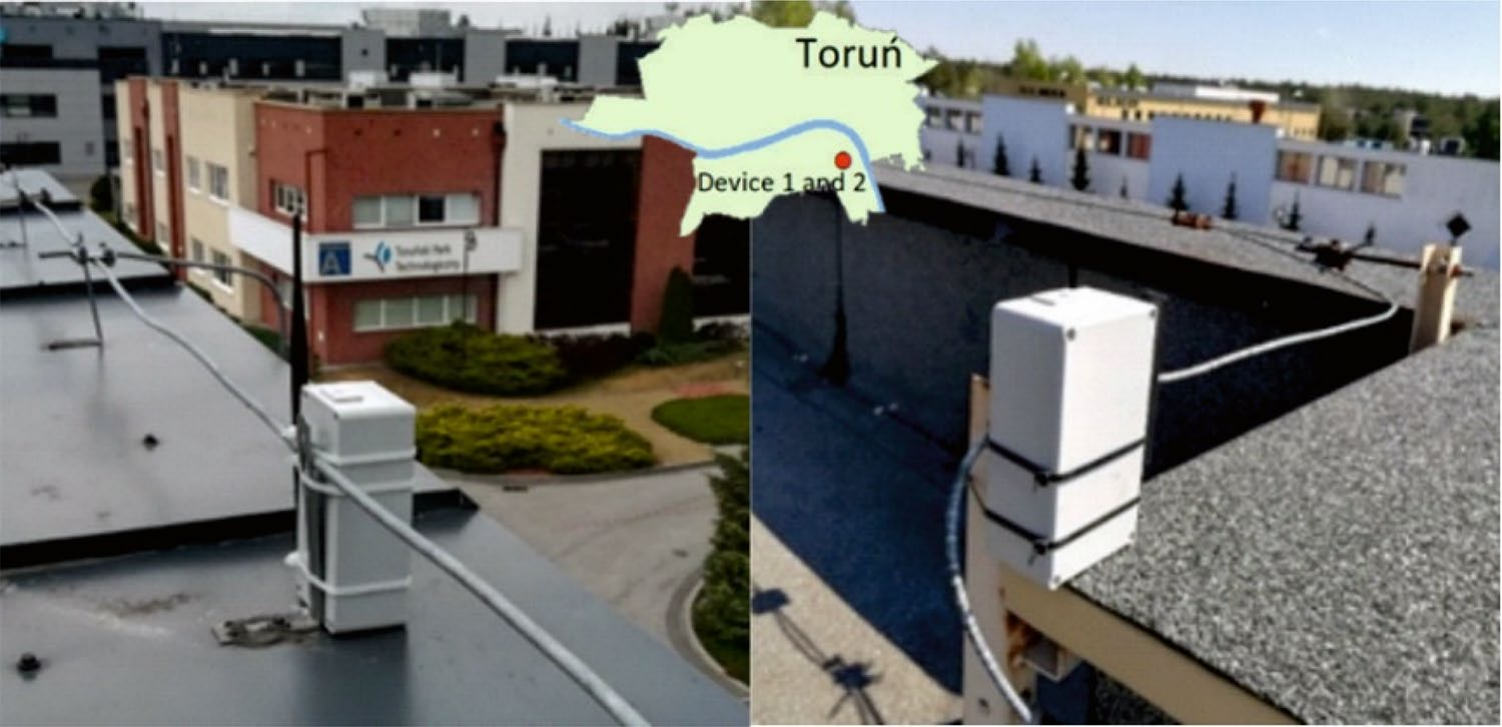
Automatic device (1 and 2) measuring the brightness of the sky, placed on one of the buildings in TARR (photo by Mieczysław Kunz).

**Figure 6:**
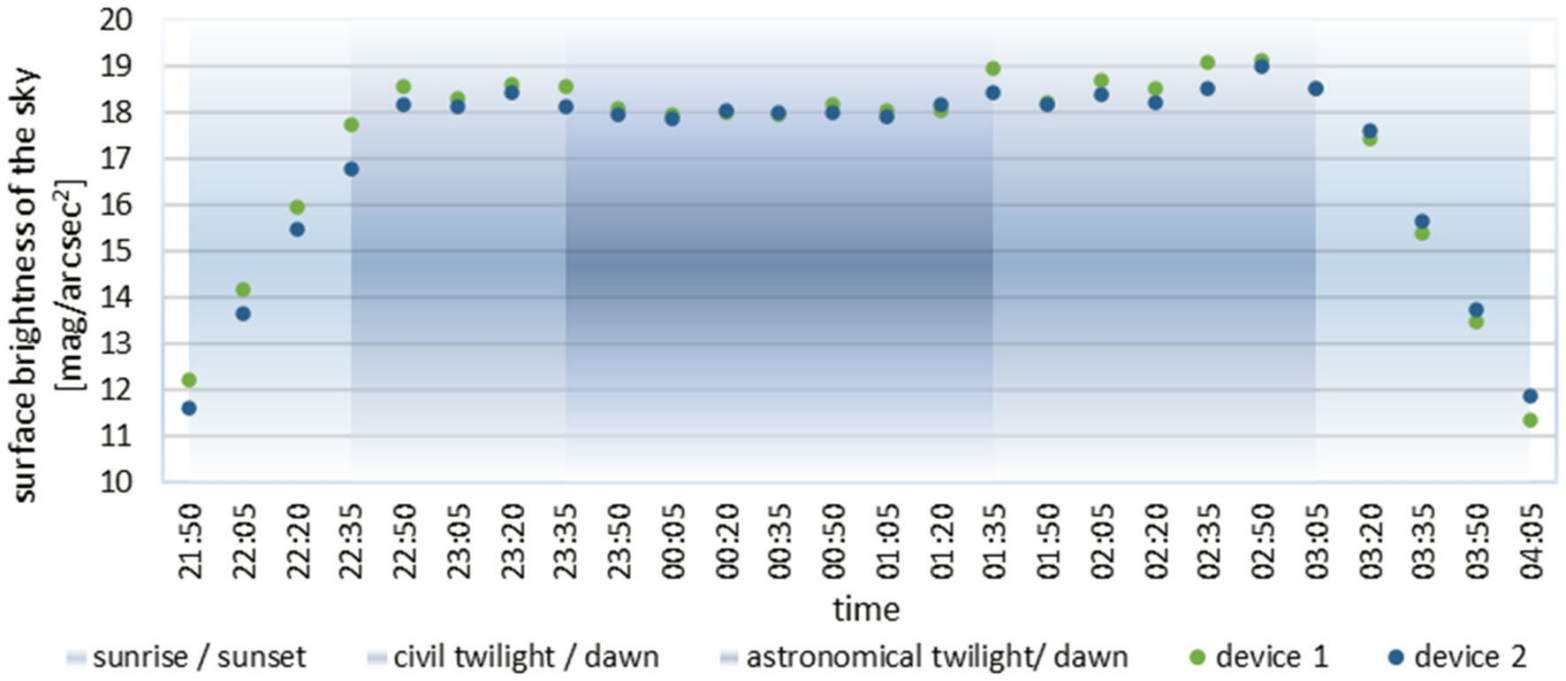
Average of measurement of sky brightness during the tests in TARR during technology demonstrator stage.

The next stage of the work performed was to check the 24 h operation mode of the devices, connectivity and the correct operation of the server where measurement data were stored. This stage was carried out at the headquarters of the Toruń Regional Development Agency (TARR) in Toruń under a signed trilateral agreement. The devices were deployed in two locations, about 100 m from each other, on the roofs of selected buildings situated within TARR (Fig. 5) and connected to the communication gateway located in the same area. The results of the pilot measurements at the TARR site are presented in Fig. 6. Through a single visualization, it shows the obtained original measurement data recorded simultaneously by the two devices, while the successive stages of the night, from sunset through civil, nautical and astronomical twilight, are shown in the background.

The analysis of the presented data shows a similarity in the curve representing the values of the phenomenon measured at both locations. The differences in their absolute values result from deploying the devices in places with slightly different local light conditions in their immediate vicinity. At the same time, during the analysis of the obtained data, the values measured at the TARR site in the 2020–2021 season using the constructed device were compared with the results obtained with the SQM photometer in the 2017–2018 season. However, weather conditions were not identical during the comparison, but to ensure similar conditions, the presented data were collected at the same time (24:00). The current location of the TARR site corresponds to the nearby RUD site, while the OKR site, used in the project carried out in the 2017–2018 season, was located near the University (NCU site). The comparison of the results from these two locations is presented in Table 2.

**Table 2:** Sample measurements from the 2017/2018 project and the new project 2020/21.

Date	2017/2018		2020/2021		
	SQM_RUD (TARR) [mag/arcsec^2^]	SQM_OKR (UMK) [mag/arcsec^2^]	TARR (device 1) [mag/arcsec^2^]	UMK (device 3) [mag/arcsec^2^]	SQM_LE (UMK) [mag/arcsec^2^]
18 November	19.07	18.66	19.14	19.13	19.12
3 April	19.17	19.02	18.54	19.06	*No data*
26 May	17.58	17.68	17.31	17.54	*No data*

The comparison shows that the results are consistent with each other. The recorded data do not differ significantly from each other, however, an in-depth comparative analysis between the measurement series will only be possible after an automatic measurement network is established throughout the city. Analyzing the data in the table, you can already notice a seasonal change in the value of the brightness of the night sky^45^. This is due to the changing length of the night during the year.

### Stage of simultaneous testing of the measuring device with the SQM LE meter

**Figure 7:**
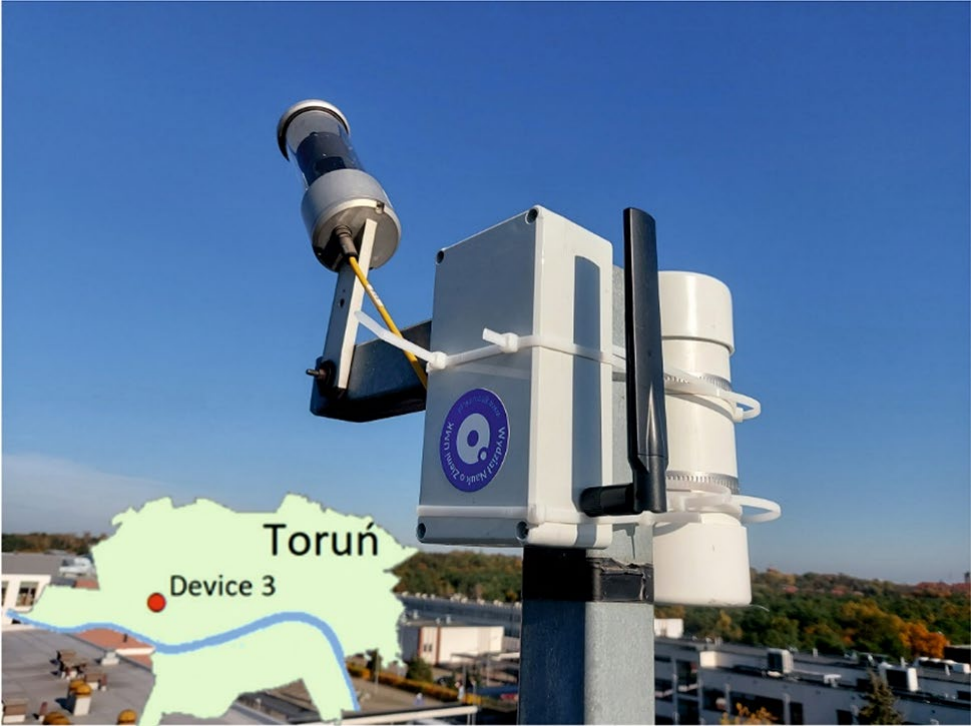
Device 3 located on the area of the Nicolaus Copernicus University in Toruń, next to the device is the SQM photometer (photo by Mieczysław Kunz).

**Figure 8:**
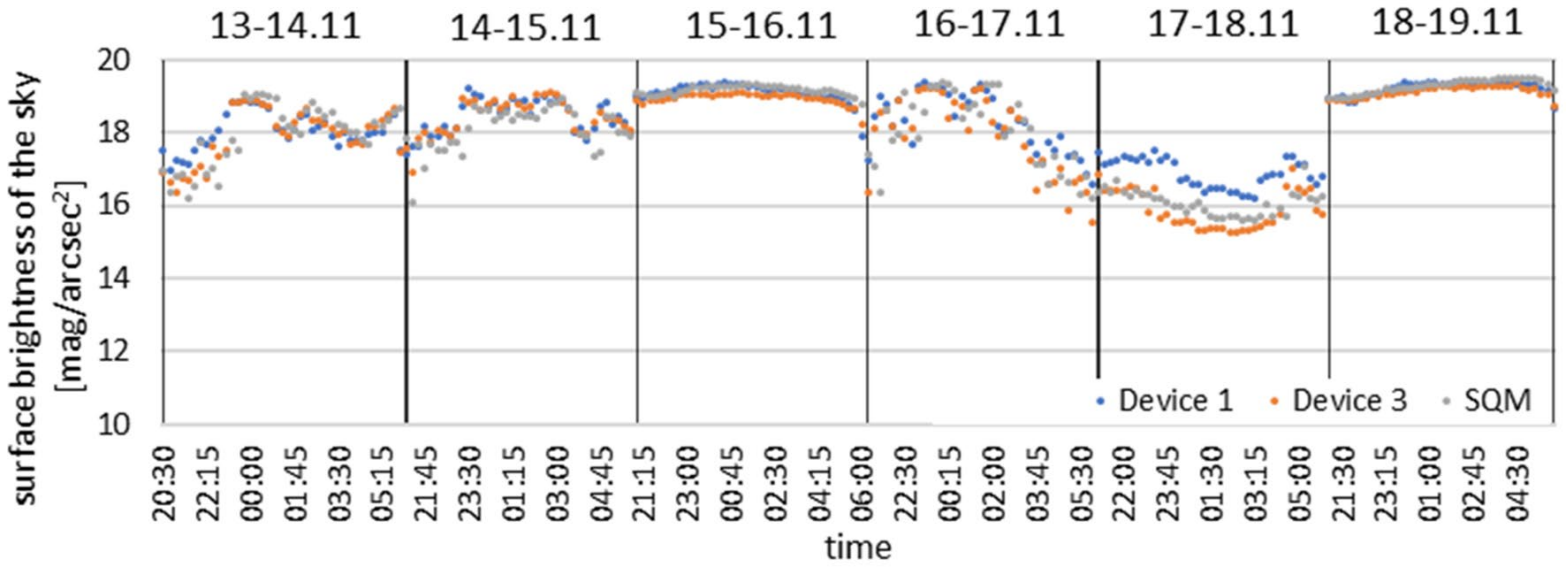
Comparison of measurements from device 1 and 3 with measurements made with the SQM photometer during consecutive nights from November 13 to 18, 2020.

After the measurement results from the first tests and the technology demonstrator stage were completed and found to be correct and reliable, another measurement device (designated as Device 3) was installed at the Nicolaus Copernicus University in Toruń. At the same time, to check again the correctness of the recorded data, a commercial photometer SQM in the LE version was installed in the same place next to that device (Fig. 7). To compare the results from both instruments (Device 3 and SQM) and additional results from one of the TARR devices (Device 1), the recorded values were converted into magnitudes and the results of the measurements taken during six consecutive nights, from 13 to 18 November 2020, were presented graphically (Fig. 8).

The graph shows high similarity between all recorded measurement data. However, it should be remembered that by using two diodes in our device, the infrared band that is present in the SQM device is compensated. Due to this difference, the results can vary statistically by about 0.2 mag/arcsec^2^. The lower results correspond to meteorological conditions when the sky was completely or heavily clouded, while fluctuations in the course of the measurement curves are observed during dynamically changing cloud cover. During a cloudless sky, the recorded data reach their maxima and the points from all the devices form a uniform, relatively horizontal, overlapping line on the graph, indistinguishable from each other, both in the course and range of the presented values.

### Stage of calibration of the set of measuring devices

**Figure 9:**
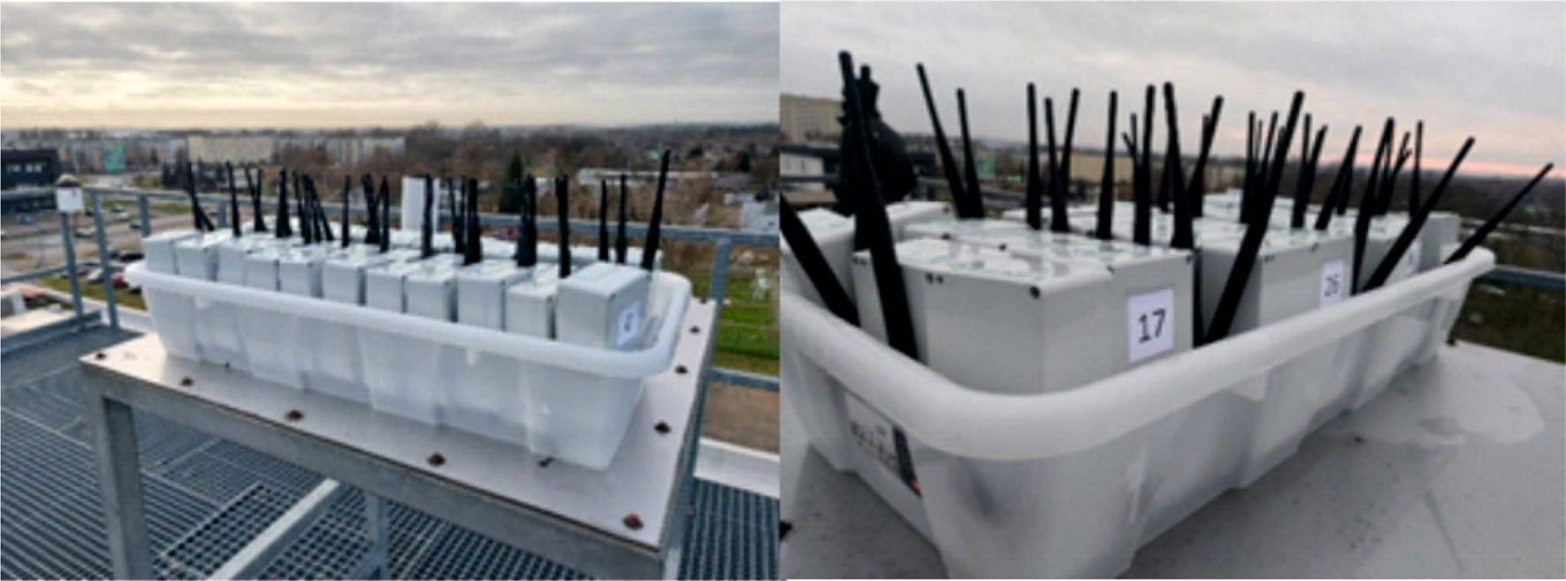
Test the cloud of 35 devices for measuring the sky brightness in winter 2020 on the terrace of the NCU Meteorological Observatory in Toruń (Poland) (﻿photo by Mieczysław Kunz).

**Figure 10:**
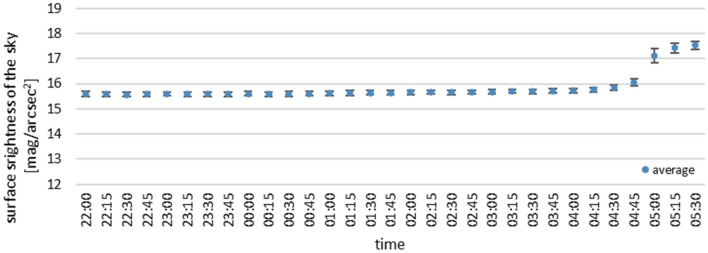
Summary of measurements from all devices during completely cloudy night of November 31 to December 1, 2020.

**Figure 11:**
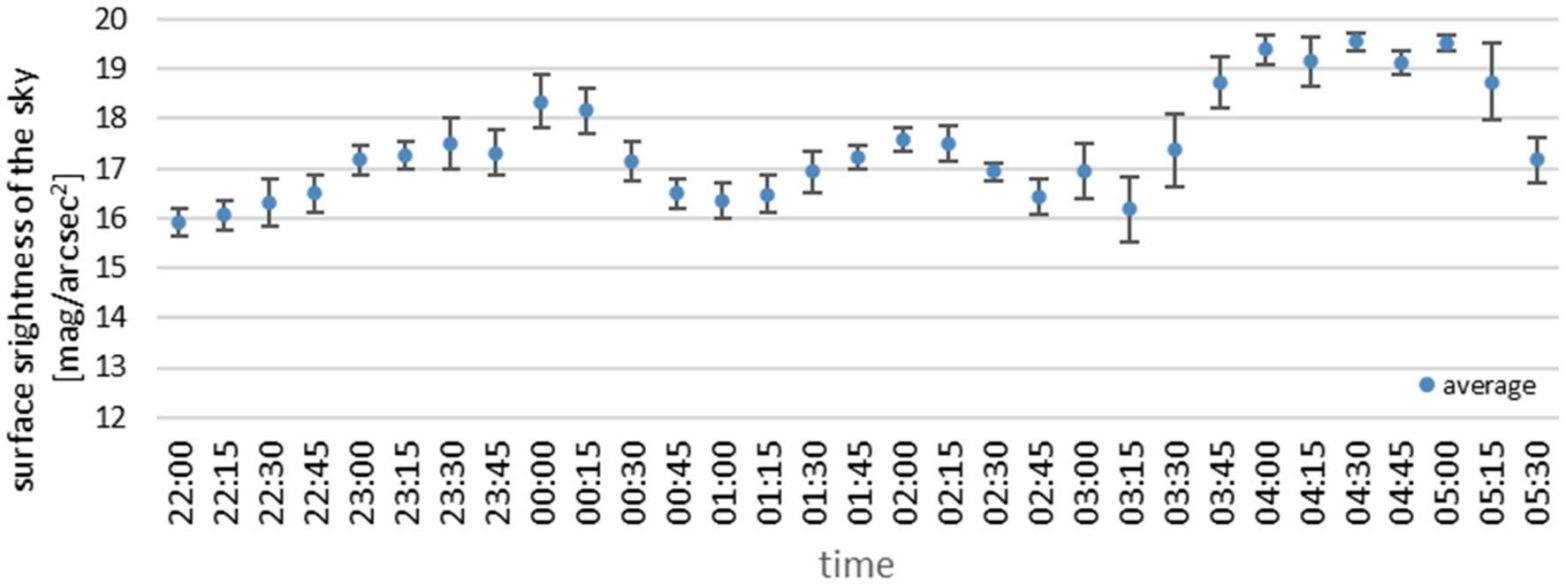
Summary of measurements from all devices during the night with variable cloudiness of December 24 to 25, 2020.

After completing all the above steps and stages of testing the first experimental measurement devices, another 35 identical units were produced, which formed the basis for the establishment and operation of a remote network to monitor artificial night light pollution in the urban space of Toruń according to the authors’ concept of implementing automatic measurements. To calibrate, i.e. to check the correctness of operation of all devices constituting the future “distributed measurement network”, they were set up simultaneously next to each other on the observation terrace of the Meteorological Observatory of the Faculty of Earth Sciences and Spatial Management of the Nicolaus Copernicus University in the direct vicinity of operating device 3 (Fig. 9). This site was selected primarily for its easy accessibility, unobstructed vision and the possibility of comparing the obtained results with the reference device (Device 3). At this stage, two test sessions were carried out during the winter season of 2020–2021, lasting continuously for one week each. The light pollution measurements of the night sky recorded by all the tested devices for an average fully cloudy night are shown in Fig. 10, and for a night with varying cloud cover in Fig. 11.

The graphs presented in Figs. 10 and 11 show high similarity of the results, including a similar course of the night measurement curve. Each graph shows the average of all measurements along with the standard deviation. The results shown in Fig. 10 were recorded under constant cloud cover, and the difference in standard deviation at each point is 0.14 mag/arcsec^2^ on average. Figure 11 shows the mean of the measurements during changing cloud cover, and here the standard deviation is about 0.3 mag/arcsec^2^. Slight differences in the course of the curve result from the fact that the measurements were only made at approximately the same time, as each device was awakened in a different part of the adopted 15 min interval.

**Figure 12:**
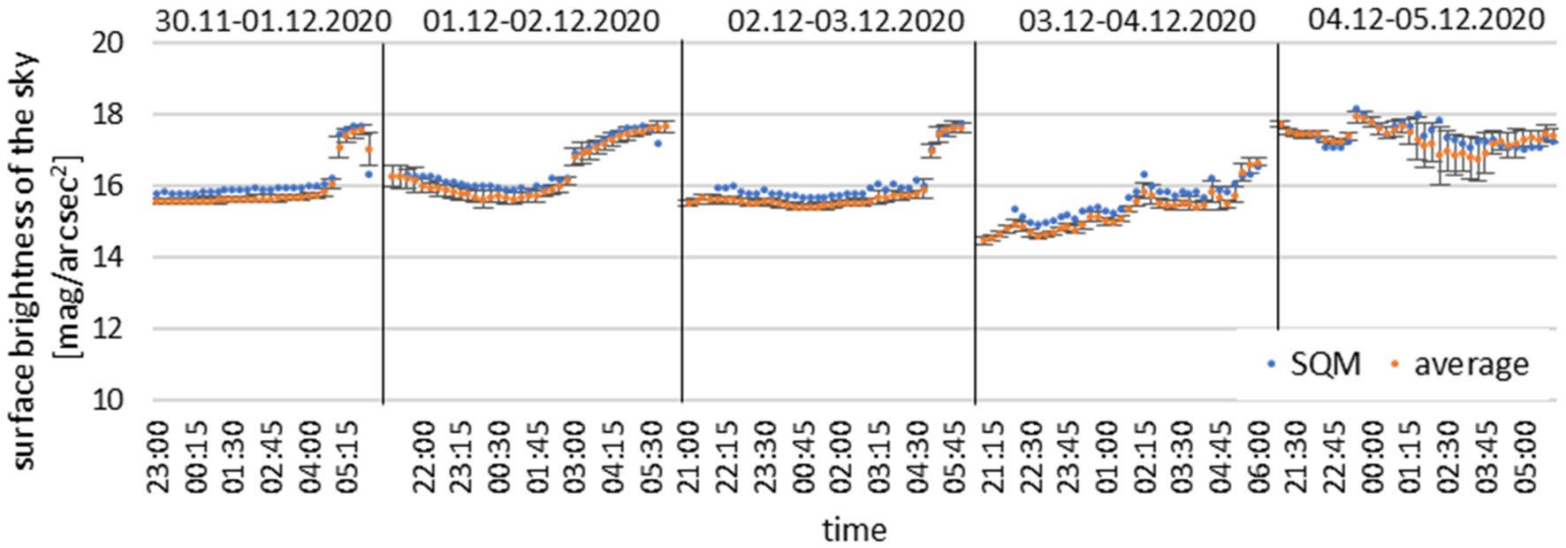
Summary of the average measurement results of all devices with the results measured with the SQM photometer.

To accurately analyse the collected data from all devices, they were averaged and presented together. The summary of the average results with the standard deviation compared to the readings from the SQM photometer at the same location are presented in Fig. 12.

The analysis of the presented graph leads to the already presented conclusions and confirms the correct operational performance, both of the prototype device and of the entire repetitive series of the prepared measuring devices. The results of the recorded measurements are consistent between the devices and correspond to the factory reference device (SQM) value. The slight differences between the readings are only due to the use of a sensor with slightly different parameters, which reduces the recorded infrared band in a different way. The differences for the minimum and maximum values for nights with varying cloud cover are due to the previously mentioned differences in the wakeup of the devices. The mean value coincides with the results from the SQM photometer, therefore the data collected by the devices can be fully comparable with the world datasets.

After all the above steps had been completed, work began on the deployment of measuring devices in Toruń and the surrounding area. At present, there are already 19 devices operating within the monitoring network and more are being deployed to form a complete measurement network evenly covering the whole city.

**Figure 13:**
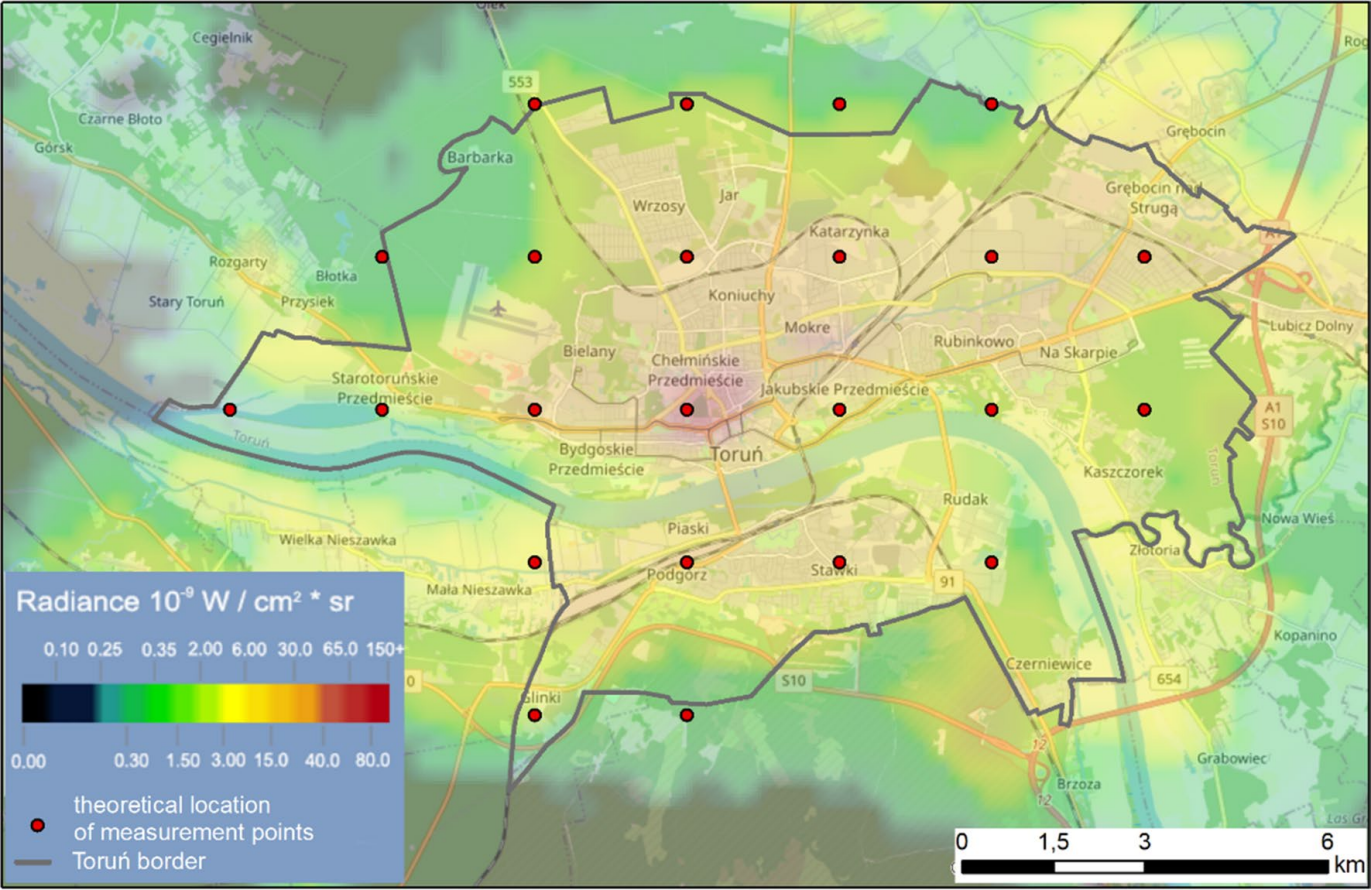
Theoretical location of measurement points. The background is the VIIRS light pollution map from 2021 at https://www.lightpollutionmap.info.

The studies carried out at the same time show that less than 40 devices are enough to cover a medium-sized city such as Toruń (about 100 km^2^)^46^. In these studies, the field of view of the device and the mean, lowest, and most common cloud base were taken into account. These simulations made it possible to determine the most advantageous distance of approximately 2.5 km. The theoretical layout of these devices is shown in Fig. 13.

## Future of the device and its use in further operations

Upon the completion of the night sky light intensity measurement network, it is planned to expand the device so that it will be possible to record also other environmental parameters, including particulate matter and concentrations of selected chemical substances. The established LoRaWAN wireless network is a good start to create multiparameter monitoring of the natural environment in urban space. The establishment of an open-access, low-cost, long-range network in an urbanised area is a value that fits into the functioning of a modern city implementing the objectives of Industrial Revolution 4.0, as well as meeting the solutions of the Smart City concept regarding Smart Environment.

## Conclusions

Targeted environmental monitoring is a necessary component of urban planning and a response to the expectations of citizens. Such targeted efforts and investments are necessary to know the extent, magnitude and intensity of all relevant parameters of human induced environmental pollution. The phenomenon of light pollution addressed in this paper is becoming more and more perceptible and a growing number of interdisciplinary studies on this issue is being conducted. They relate both to understanding the causes and extent of this negative phenomenon, as well as to its impact on all living organisms, including primarily human beings. Therefore, it seems necessary to monitor it in a continuous manner. The developed original device for sky brightness measurement, operating in a distributed measurement structure, is a proposal for measuring light smog in urban areas. The network for monitoring the night sky brightness in the area of Toruń, proposed conceptually and implemented, has successfully passed all subsequent efficiency tests, including measurement, operation, communication and data archiving. The measurement data obtained by the constructed remote automatic recording device are accurate and corresponding in value to the data obtained by a commercial photometer available on the market. This gives a full and complementary possibility of comparing the registration by the constructed device with the results obtained during measurements carried out in other parts of the world or in relation to archival values. Further steps are planned to expand and adapt the monitoring network established in Toruń for subsequent projects. The design of the device allows it to be adapted to measure other environmental parameters. There is also great flexibility in configuring the equipment for new tasks and challenges that may arise during operation.

## Data Availability

All data analysed during this study are included in this article. If anyone wants the datasets they are available from the corresponding author on request.
